# IMATAC imputes single-cell ATAC-seq data by deep hierarchical network with denoising autoencoder

**DOI:** 10.1093/bib/bbaf515

**Published:** 2025-09-29

**Authors:** Yao Li, Hongqiang Lyu, Kexin Li, Yuan Liu, Xinman Zhang, Ze Liu, Pengcheng Jing, Peng Han

**Affiliations:** Faculty of Electronic and Information Engineering, School of Automation Science and Engineering, Xi'an Jiaotong University, No. 28 Xianning West Road, Beilin District, Xi'an, Shaanxi 710049, China; Faculty of Electronic and Information Engineering, School of Automation Science and Engineering, Xi'an Jiaotong University, No. 28 Xianning West Road, Beilin District, Xi'an, Shaanxi 710049, China; Faculty of Electronic and Information Engineering, School of Automation Science and Engineering, Xi'an Jiaotong University, No. 28 Xianning West Road, Beilin District, Xi'an, Shaanxi 710049, China; Faculty of Electronic and Information Engineering, School of Automation Science and Engineering, Xi'an Jiaotong University, No. 28 Xianning West Road, Beilin District, Xi'an, Shaanxi 710049, China; Faculty of Electronic and Information Engineering, School of Automation Science and Engineering, Xi'an Jiaotong University, No. 28 Xianning West Road, Beilin District, Xi'an, Shaanxi 710049, China; College of Water Resources and Architectural Engineering, Northwest A&F University, No. 3 Taicheng Road, Yangling District, Xianyang, Shaanxi 712100, China; Faculty of Electronic and Information Engineering, School of Automation Science and Engineering, Xi'an Jiaotong University, No. 28 Xianning West Road, Beilin District, Xi'an, Shaanxi 710049, China; Department of Otorhinolaryngology—Head and Neck Surgery, The First Affiliated Hospital, Xi'an Jiaotong University, No. 277 West Yanta Road, Yanta District, Xi'an, Shaanxi 710061, China

**Keywords:** single-cell ATAC-seq, imputation, hierarchical network, denoising autoencoder

## Abstract

Single-cell ATAC-seq (scATAC-seq) technology allows the interrogation of chromatin accessibility of individual cells. Dropout events occur while the sequencing data signals at some bona fide chromatin sites of individuals are not captured, and the curse of these dropouts in scATAC-seq data inevitably hinders downstream analysis. It remains a challenge to impute scATAC-seq data due to its high dimensionality, sparsity, and near-binarization properties. Herein, we propose IMATAC, a deep hierarchical network with denoising autoencoder for imputing scATAC-seq data in the form of peak by cell. The network embeds scATAC-seq data into a latent space by a deep hierarchical architecture at two different levels, including bottom level for local details and top level for global information, that helps to characterize the high-dimensional sparse scATAC-seq data. Besides, it is encouraged to learn to reconstruct the original scATAC-seq data from an artificially corrupted version through a denoising autoencoder, so as to acquire an ability to recover the missing values primarily relying on the cells under the same population with the help of a parallel multi-classifier. Using simulated and experimental data, the performance of IMATAC is demonstrated by a comparative analysis with the other competing methods. The results suggest that our method can achieve lower imputation errors, and benefit the downstream analysis, including heterogeneous clustering, differential analysis, and regulatory element discovery. Besides, the contributions of several important network modules in our IMATAC are investigated, and how well it can separate the dropout zeros from biological zeros are discussed.

## Introduction

Accessible regions within chromatin host a network of complex interplays among gene regulatory machineries, such as *cis-*regulatory elements, Transcription Factors (TFs), cofactors, and chromatin remodelers [1, 2]. To profile chromatin accessibility, a number of technologies have been developed, including DNase-seq [3], MNase-seq [4], FAIRE-seq [5], ATAC-seq [6], NOMe-seq [7], and MPE-seq [8]. Among them, ATAC-seq is an ensemble measure of open chromatin that uses prokaryotic Tn5 transposase to tag regulatory regions by inserting sequencing adapters into accessible regions of genome, and is regarded as the most widely used one owing to its lower input material requirements and shorter assay time [9, 10]. Recent advances in ATAC-seq technology have allowed interrogation of chromatin accessibility at single-cell level [11, 12]. In single-cell ATAC-seq (scATAC-seq), adaptors are integrated into accessible chromatin regions of individual cells with the help of a programmable microfluidics platform following a series of optimized procedures for this task, and a coordinate-based count matrix is expected to be yielded eventually in two optional forms, including peak by cell and bin by cell. The former takes accessible peaks called computationally as genomic regions of interest and is much more popular, while the latter focuses on genomic bins with fixed-length [13, 14]. Beyond ATAC-seq at bulk level, scATAC-seq has an ability to deconvolve heterogeneous cellular mixtures in terms of chromatin accessibility, and provides insights into cell-to-cell variation within a complex tissue [11, 13]. However, in scATAC-seq experiment, each open chromatin site of a diploid-genome cell has only one or two opportunities to be captured. Typically, just a few thousand distinct reads are available per cell in contrast with many thousands of possible open positions, resulting in a lack of sequencing data signals at some bona fide open chromatin sites of the cell. That makes some peaks observed at a low or moderate count level in one cell but not detected in another cell under the same population, known as dropout events [15, 16]. The curse of these dropout events inevitably hinders the downstream analysis of scATAC-seq data.

Imputation methods are devoted to addressing dropout events in the analysis of single-cell omics, and the design of imputation methods for scATAC-seq data in the form of peak by cell needs to consider two issues. One is high dimensionality. The number of peaks in scATAC-seq data on a genome scale can reach more than 100 K or even higher, which is much larger than that of genes in scRNA-seq data [17]. The other is sparsity. scATAC-seq typically produces only 3%–7% of non-zero counts in peak by cell matrix, which is distinct from 10%–30% of non-zero values for scRNA-seq data, since scATAC-seq profiles DNA not RNA, the much lower copy number (one or two) per diploid cell results in an inherent sparsity and near-binarization [14, 18, 19]. Currently, there are several existing methods devoted to the imputation of peak count vectors of individual cells, including SCALE [20], scOpen [21], SAILER [22], peakVI [23], and scCASE [24]. Among them, SCALE provides a variational autoencoder to learn the latent features that characterize inputs, the features in the latent space are assumed to follow a mixture of Gaussian distributions, and the imputed scATAC-seq data can be generated by the decoder of this network. scOpen performs a frequency-inverse document frequency transformation on the binarized peak by cell matrix, and a regularized non-negative matrix factorization is employed to deal with the sparsity problem and reconstruct the imputed scATAC-seq data. SAILER and peakVI also take advantage of variational autoencoder to embed and reconstruct scATAC-seq data, just like SCALE. The difference is that the former imposes confounding factors, including read depth and batch effects, to enhance the independence of the learned latent representations. While the latter takes library size and region-specific biases into account, and pays more attention to differential analysis rather than imputation. As for the last method scCASE, beyond the regularized non-negative matrix factorization, an iterative updating of cell-to-cell similarity matrix is simultaneously incorporated to impute scATAC-seq data. Nevertheless, to the best of our knowledge, there is still some room for improvement, such as distinction between dropout zero and biological zero, overcoming of cellular heterogeneity, and consideration of multimodal data. Besides, considering that the imputation methods for scRNA-seq data have made breakthroughs in recent years, how well these methods can be applied to scATAC-seq data has also been investigated [15], including MAGIC [25], SAVER [26], scImpute [27], deepImpute [28], PRIME [29], bayNorm [30], and knn-smoothing [31]. The analysis results indicate that these methods can indeed be applied to scATAC-seq data in the implementation, but their performance on different scATAC-seq datasets varies greatly. Even though, the methods for scRNA-seq data are still sub-optimal options while standing together with those designed for scATAC-seq data, since the two kinds of data differ from each other in many aspects, including dimensionality, sparsity, and distribution. As the dropout events of high-dimensional sparse scATAC-seq data continue to be a bottleneck while the measurable cell counts keep increasing, the demand for scATAC-seq imputation methods keeps rising.

In this paper, we present IMATAC, a deep hierarchical network with denoising autoencoder to impute scATAC-seq data. Beyond the existing imputation methods for scATAC-seq data, IMATAC addresses the following two issues. One is a hierarchical network architecture to embed high-dimensional sparse data. The normalized peak count vector for each cell is embedded by a deep hierarchical network into a latent space at two different levels, including bottom level for local details and top level for global information. This hierarchical architecture benefits the comprehensive characterization of high-dimensional sparse data. The other one is a denoising autoencoder to learn to recover missing data. A portion of peaks are artificially masked for each cell before being fed into autoencoder, the network is encouraged to predict the removed content as much as possible by considering the same populations of cells with the help of a multi-classifier, so as to acquire an ability to recover the masked values, and this ability helps effectively respond to dropout events. Using both simulated and experimental scATAC-seq data, these two issues are demonstrated by a comparative analysis with the other state-of-the-art methods, including SCALE, scOpen, SAILER, peakVI, scCASE, and scDenoise [32]. The results tell that our IMATAC can produce lower imputation errors in terms of mean squared error (MSE) and meta-cell distance, enhance the cluster pattern of heterogeneous single cells, benefit the biological analysis of differential peaks, and help the discovery of regulatory elements. In addition, we also discuss whether the hierarchical network architecture, artificial masking, and multi-classifier in IMATAC are conducive to the recovery of missing data, and to what extent our method can separate the dropout zeros from biological zeros. In summary, the contributions of this paper are threefold. (i) A new hierarchical network with two levels is proposed to handle high-dimensional sparse scATAC-seq data. (ii) A denoising autoencoder is introduced to reconstruct scATAC-seq data from an artificially corrupted version, so as to acquire an ability to recover the missing values by dropout events. (iii) A dual-task 1D-CNN network consisting of a denoising autoencoder and a multi-classifier is constructed to encourage the imputation of scATAC-seq data from the same populations of cells.

## Materials and methods

### Dataset

Both simulated and experimental scATAC-seq data are involved in this study to evaluate the performance of IMATAC. For simulated data, a scATAC-seq dataset of five cell types (A, B, C, D, and E) was generated in the form of peak by cell matrix with the help of SimCAS [33]. A total of 2500 cells were produced using default configurations, with 80 000 peaks for each cell. Besides, a total of six scATAC-seq datasets with other considerations, including different levels of noise (level 1, level 2, and level 3) and different degrees of sparsity (0.4, 0.5, and 0.6), were also simulated based on this (Supplementary methods). Then a portion of the peaks for each cell were artificially masked at random to obtain a corrupted version of the data with dropout events. For experimental data, there are four datasets involved, including Buenrostro2018 [34], GM12878/HL60 [14], InSilico [11], and Splenocyte [35]. Among them, Buenrostro2018 dataset is from CD34+ human bone marrow, consists of 2034 hematopoietic cells that were profiled and FACS-sorted from 10-cell populations, including hematopoietic stem cells (HSCs), multipotent progenitors (MPPs), lymphoid-primed multipotent progenitors, common myeloid progenitors, granulocyte-macrophage progenitors, megakaryocyte-erythroid progenitors (MEPs), common lymphoid progenitors, monocytes (mono), uterine natural killers, and plasmacytoid dendritic cells. To have the peak by cell matrix, a BAM file was downloaded, followed by a peak calling with MACS2 [36], and a matrix over the union of peaks was generated by counting the number of reads from individual cells that overlap the union peaks with the help of bedtools [37]. GM12878/HL60 and InSilico datasets contain 1987 and 1377 cells, respectively, and are divided into two and six cell populations correspondingly. The former is composed of cell types GM12878 and HL60, and the latter has cell types H1ESC, TF-1, BJ, HL-60, GM12878, and K562. The relevant scATAC-seq data can be downloaded from NCBI (GSE109828 and GSE65360) or Zenodo (3984189). Splenocyte dataset is derived from a mixture of 3166 mouse splenocytes (after red blood cell removal). It consists of 12-cell populations, including CD27-_Natural_Killer, Macrophage, Memory_CD8_T, CD27 + _Natural_Killer, Regulatory_T, Dendritic_cell, Granulocyte, Follicular_B, Naive_CD4_T, Marginal_Zone_B, Transitional_B, and Naive_CD8_T. And the corresponding scATAC-seq data is available from ArrayExpress (E-MTAB-6714) or Zenodo (3984189).

### Overview of IMATAC

**Figure 1:**
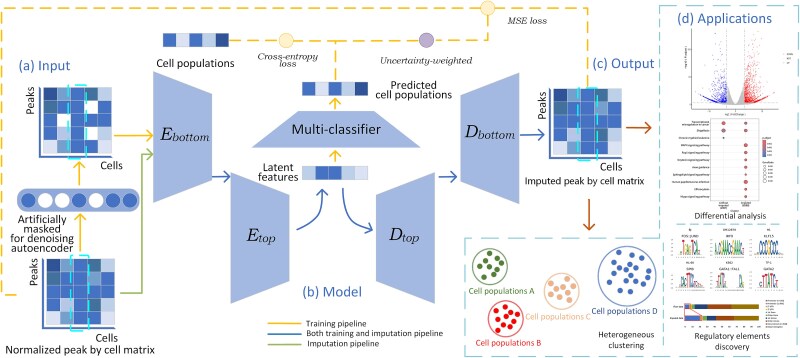
Overview of IMATAC. IMATAC is a deep hierarchical convolution network with a denoising autoencoder for the imputation of scATAC-seq data. (a) Input. A portion of peaks are first artificially masked at random for each cell, and a corrupted version of peak by cell matrix is taken as input. (b) Model. The input is embedded into a latent space through a hierarchical encoder composed of ${E}_{bottom}$ and ${E}_{top}$, the cells in the latent space are grouped with the help of a multi-classifier, and the original data is reconstructed by a corresponding hierarchical decoder denoted as ${D}_{top}$ and ${D}_{bottom}$. The loss function consists of two parts, including MSE loss for the denoising autoencoder and cross-entropy loss for the multi-classifier, which are weighed by considering the homoscedastic uncertainty of each part. After training, IMATAC acquires an ability to recover the missing content by dropout events, primarily relying on the cells under the same population. (c) Output. The reconstructed peak by cell matrix is regarded as output with the missing values imputed as much as possible. (d) Applications. How well our method benefits the downstream analysis is discussed, including heterogeneous clustering, differential analysis, and regulatory element discovery. It is worth noting that the artificial masking is part of the denoising autoencoder, and the cell populations can be determined according to cell types, cell-cycle states, cell differentiation stages, and even clustering labels once the first few are unavailable.

IMATAC is a deep hierarchical network with denoising autoencoder designed for the imputation of scATAC-seq data. It takes a peak count vector for each cell as input, and outputs an imputed one in which the missing values by dropout events can be recovered (Fig. 1). In IMATAC, the training procedure starts with an artificial masking, where a portion of the peaks are artificially masked at random for each cell to obtain a corrupted version of data. Then, the corrupted data is fed into a hierarchical encoder composed of ${E}_{bottom}$ and ${E}_{top}$, and transformed into a latent space at two levels, including bottom level and top level, which are responsible for representing local details and global information, respectively. Meanwhile, the cells in the latent space can be divided into groups as close to their populations as possible with the help of a multi-classifier. And finally, the output data is generated through a corresponding hierarchical decoder, denoted as ${D}_{top}$ and ${D}_{bottom}$, with the masked peaks recovered as much as possible. That makes IMATAC learn to reconstruct the input scATAC-seq data from an artificially corrupted version through its deep hierarchical network, so as to acquire an ability to recover missing content primarily relying on the cells under the same population. After training, in the imputation procedure, IMATAC can be used to predict new plausible values for dropouts in scATAC-seq data. The details can be found below.

### Normalization

A normalization preprocessing is involved to make that the values in peak by cell matrix are on the same scale. That is beneficial for the network to learn the laws underling a great number of training samples. Let ${\boldsymbol{X}}^{\prime}\in{\mathbb{R}}^{m\times n}$ stands for a raw peak by cell matrix, where $m$ represents the number of peaks, $n$ indicates the number of single cells, ${X}_{ij}^{\prime }$ is the counts of reads falling into peak $i,1\le i\le m$ for cell $j,1\le j\le n$. In the implementation, the element ${X}_{ij}^{\prime }$ will be filtered out first as an outlier when its value is extremely high, followed by a transformation that is implemented on a log scale to obtain the normalized matrix$\boldsymbol{X}\in{\mathbb{R}}^{m\times n}$:


(1) \begin{equation*} {X}_{ij}=\frac{lo{g}_2\left({X}_{ij}^{\prime }+1\right)}{\underset{1\le j\le n}{\max } lo{g}_2\left({X}_{ij}^{\prime }+1\right)} \end{equation*}

### Network architecture

IMATAC is a dual-task 1D-CNN network consisting of a denoising autoencoder and a multi-classifier, where the former serves the reconstruction task, and the latter is dedicated to the classification task. For the reconstruction task, a denoising autoencoder is designed to embed and reconstruct the normalized peak count vector for each cell through a set of hierarchical encoders and decoders at two different levels, including bottom level and top level. It starts with a bottom encoder ${E}_{bottom}$, which takes the normalized peak count vector for a cell, namely a column in peak by cell matrix $\boldsymbol{X}$, as input, and gives out its local detail features by means of two 1D convolutional layers. The first layer utilizes $1\times 4$ convolution kernel with a stride of $2$ to extract local features, followed by dropout regularization and ReLU activation. And the second layer performs a channel fusion using kernel size $1\times 3$ and stride $1$. Combined with ${E}_{bottom}$, the top encoder ${E}_{top}$ accepts the output of ${E}_{bottom}$, and the global information can be retrieved through three 1D convolutional layers. ${E}_{top}$ is constructed in the same way as ${E}_{bottom}$ with the exception that the first layer is duplicated. It can be seen that there is a preliminary feature extraction from input at bottom level, so that the local details can be captured by ${E}_{bottom}$. As the local details move from bottom level to the top level, they are further combined and aggregated to form the top level patterns, so that the global information can be captured by ${E}_{top}$. With the help of this hierarchical encoder, the peak count vector is embedded into a latent space. Then a corresponding hierarchical decoder with a mirror-symmetric transposed convolutional architecture, including ${D}_{top}$ and ${D}_{bottom}$, is devoted to the reconstruction of this vector from the latent space. It is worth noting that the outputs of ${E}_{top}$ and ${E}_{bottom}$ will be concatenated together before being fed into ${D}_{bottom}$. For the classification task, a multi-classifier is designed to divide the cells in the latent space into groups as close to their populations as possible through two layers. Specifically, the first fully connected layer accepts the latent features of each cell followed by batch normalization and ReLU activation function, and subsequently comes the second fully connected layer with SoftMax activation function, which enables the network to distribute the probabilities into $q$ different populations, and the one with the highest probability value is considered as the most likely population that the cell belongs to. The cell populations can be determined according to cell types, cell-cycle states, and cell differentiation stages. Furthermore, once the above aren't available, these populations can also be decided by clustering algorithms [19, 38–40].

The proposed IMATAC has differences compared with the other six competing methods, including SCALE, scOpen, SAILER, peakVI, scCASE, and scDenoise. First of all, our method is completely out of the same category as scOpen and scCASE, since they are not deep-learning-based methods but matrix-based ones, where a regularized non-negative matrix factorization is employed to decompose and reconstruct sparsity matrix. As for the remaining four methods, our IMATAC does have the same encoder-decoder framework as them, such as the ordinary autoencoder in SCdenoise and the variational autoencoders in SCALE, SAILER, and PeakVI. Especially, SCdenoise has a multi-classifier in parallel with an autoencoder, which is very close to the dual-task learning of our method. Even so, as far as we know, our IMATAC is beyond the four deep-learning-based methods in terms of hierarchical network with two levels and denoising autoencoder, which are believed to be beneficial for the embedding of high-dimensional sparse scATAC-seq data and the learning of an ability to recover the missing values.

### Uncertainty-weighted loss

Two main loss functions are involved to conduct the learning of the two parallel tasks, including the reconstruction of scATAC-seq data via the denoising autoencoder and classification of single cells via the multi-classifier. For the former, an MSE loss function is employed to measure the average squared difference between the decoder output and original input, which is given by:


(2) \begin{equation*} {L}_{MSE}=\frac{1}{n}{\sum}_{j=1}^n{\left\Vert D\left(E\left({X}_{\bullet j}\right)\right)-{X}_{\bullet j}\right\Vert}^2 \end{equation*}

where $E$ and $D$ indicates the hierarchical encoder and decoder, respectively, and ${X}_{\bullet j}$ denotes the normalized peak count vector for the ${j}_{th}$ cell, i.e. the ${j}_{th}$ column in peak by cell matrix $\boldsymbol{X}$. For the later, a cross-entropy loss function is used to quantify how well the predictions of multi-classifier match the ground truth labels of cell populations, which is defined as:


(3) \begin{equation*} {L}_{CE}=-\frac{1}{n}{\sum}_{i=1}^n{\sum}_{j=1}^q{\hat{c}}_{ij}\log \left({c}_{ij}\right) \end{equation*}

where ${\hat{c}}_{ij}$ refers to the ground truth one-hot encoded value for the ${i}_{th}$ cell under the ${j}_{th}$ population, and ${c}_{i,j}$ denotes the predicted probability that the ${i}_{th}$ cell may be under the ${j}_{th}$ population.

During the training process, the denoising autoencoder and multi-classifier influence each other, since they share a common hierarchical encoder. Specifically, the denoising autoencoder pushes the encoder to extract meaningful latent features free from noises that help to reconstruct the original signals, while the multi-classifier encourages the encoder to learn low-dimensional latent presentations of single cells that are discriminative between different populations. To support the dual-task training, the two loss functions above are weighed by considering the homoscedastic uncertainty of each task. Given that the errors by the denoising autoencoder and multi-classifier follow a Gaussian distribution, IMATAC is eventually trained by minimizing an uncertainty-weighted loss:


(4) \begin{equation*} L=\frac{1}{\sigma_1^2}{L}_{MSE}+\frac{1}{\sigma_2^2}{L}_{CE}+2\mathit{\log}{\sigma}_1+2\mathit{\log}{\sigma}_2 \end{equation*}

where ${\sigma}_1$ and ${\sigma}_2$ are the variances of the errors by denoising autoencoder and multi-classifier, respectively, and the two loss functions, including ${L}_{MSE}$ and ${L}_{CE}$, are eventually combined into a comprehensive uncertainty-weighted loss $L$, to simultaneously optimize the objectives of the two tasks with the help of Adam [41].

## Results

### Results on simulated data

**Figure 2:**
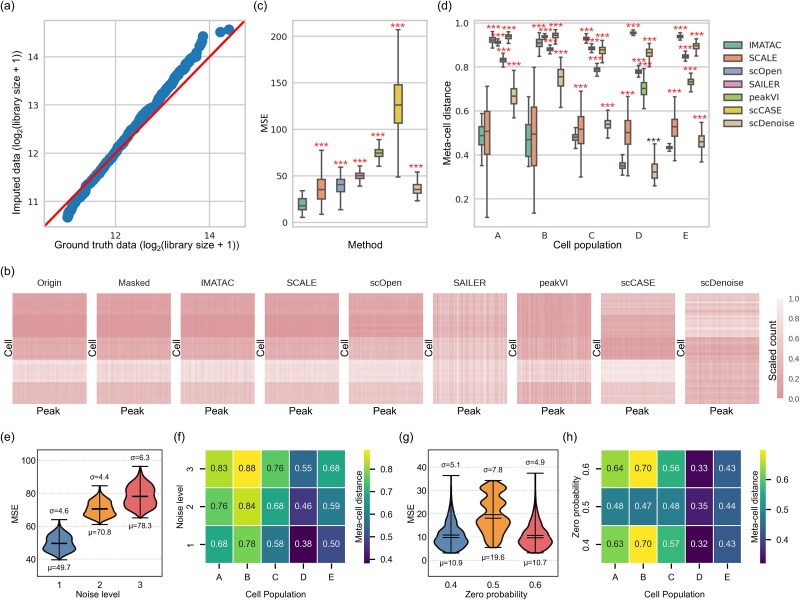
Results on simulated scATAC-seq data. The performance of IMATAC is investigated on simulated scATAC-seq data of five cell types (A, B, C, D, and E) at a masking rate of 40%, and compared with that of the other six competing methods, including SCALE, scOpen, SAILER, peakVI, scCASE, and scDenoise. The statistical significance is calculated using a one-tailed t-test, the ranges of p-values are marked with different numbers of asterisks considering the limited space, i.e. one asterisk for [0.05, 0.01), two asterisks for [0.01, 0.001), and three for [0.001, −∞), and the directions are indicated by red and black colors, respectively. (a) Scatter plot of library size between ground truth data and imputed data by IMATAC. (b) Heatmaps of peak by cell matrices, including original, masked, and imputed by IMATAC and the other six methods. The high-dimensional matrices have been reduced for visualization by merging surrounding counts. (c) Box plot of imputation error in terms of MSE by IMATAC and the other six methods. (d) Box plot of imputation error in terms of meta-cell distance by IMATAC and the other six methods. (e) Violin plot of imputation error in terms of MSE by IMATAC across different levels of noise (level 1, level 2, and level 3). (f) Heatmap of imputation error in terms of meta-cell distance by IMATAC across different levels of noise. (g) Violin plot of imputation error in terms of MSE by IMATAC across different degrees of sparsity (0.4, 0.5, and 0.6). (h) Heatmap of imputation error in terms of meta-cell distance by IMATAC across different degrees of sparsity.

The simulated scATAC-seq data of five cell types (A, B, C, D, and E) at three different masking rates (20%, 40%, and 60%) was fed into IMATAC to investigate its performance. Firstly, the library sizes for individual cells were calculated, and they are compared between the ground truth data and imputed data at a masking rate of 40% (Fig. 2a). In general, most scatter points are very close to the ideal line, indicating that IMATAC can recover the masked content on a reasonable scale. Then, the heatmaps of a series of peak by cell matrices, including original, masked, and imputed by IMATAC, SCALE, scOpen, SAILER, peakVI, scCASE, and scDenoise, are visualized (Fig. 2b). Intuitively, most of these methods, especially IMATAC, SCALE, and scOpen, can provide imputed matrices with a pattern very similar to the original one, demonstrating their performance in recovering the masked data. Taking a step forward, this performance of IMATAC is scored by comparing the ground truth and imputed data using MSE, and compared with that of the six competing methods (Fig. 2c). It can be seen that IMATAC has the lowest values with a mean of 19.33 ± 7.78, outperforming the other six competing methods significantly (*p*-value ≤0.001). Besides, the distance between the imputed peak count vector for each cell and its corresponding meta-cell is also calculated (Fig. 2d), where the meta-cell is defined as the average of a specific cell population [15]. With respect to this metric, our IMATAC also has a considerable performance, with the mean distances of 0.48 ± 0.06, 0.47 ± 0.08, 0.48 ± 0.02, 0.35 ± 0.02, and 0.44 ± 0.01 for cell types A, B, C, D, and E, respectively. Compared with the six competing methods, our method can substantially achieve the lowest values across different cell types, except for SCALE and scDenoise, whose distances are competitive in some cases, but the former often has a larger degree of dispersion, and the latter fluctuates greatly. It suggests that IMATAC has the ability to take information from the same populations of cells, which may benefit from the multi-classifier in the network architecture. In addition, at the masking rates of 20% and 60%, the comparative analysis is also conducted in the same way (Supplementary Figs. S1–S2). Generally, IMATAC still has the lowest imputation errors in terms of MSE and meta-cell distance at the two masking rates in most cases. The details can be found in Supplementary Tables S2–S3. Furthermore, we further investigated how different levels of noise (level 1, level 2, and level 3) and different degrees of sparsity (0.4, 0.5, and 0.6) in scATAC-seq data affect the performance of IMATAC (Fig. 2e–h). As the noise intensity increases, the values of MSE and meta-cell distance also increase as expected, but show a weakening upward trend. As for the sparsity, there is no specific pattern presented while the probability of zeros in peak by cell matrix changes, indicating the robustness of our method in the presence of different degrees of sparsity.

### IMATAC retains and enhances the cluster patterns of single cells

**Figure 3:**
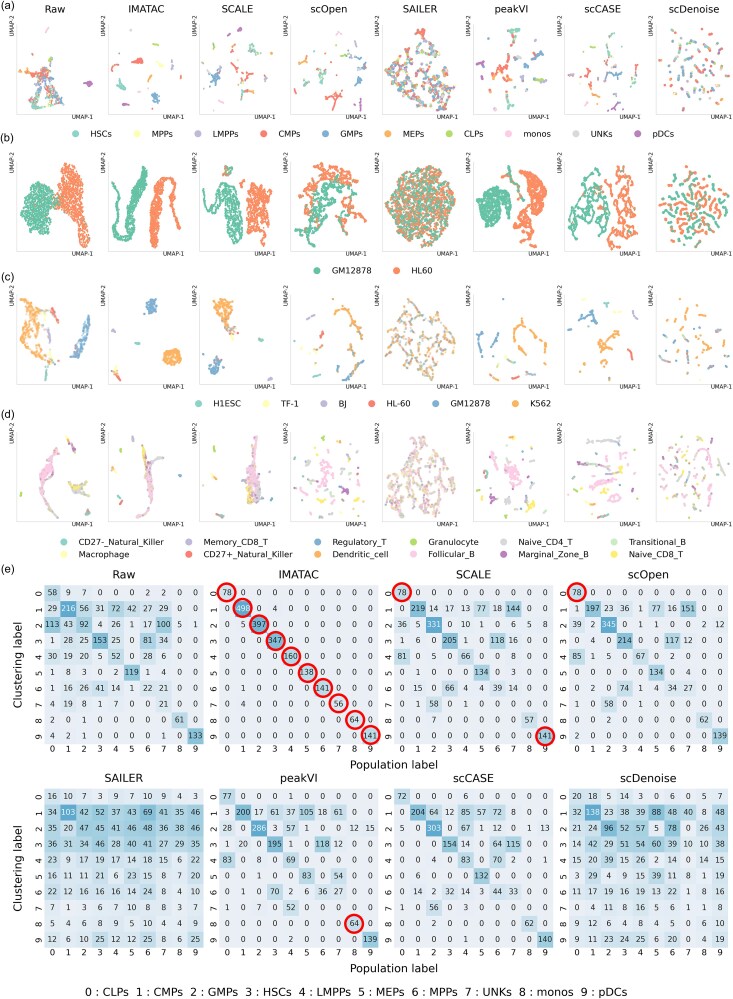
Clustering analysis of imputed scATAC-seq data. Single cells are embedded by combining PCA and UMAP, and clustered using K-means on scATAC-seq data imputed by IMATAC and the other six competing methods, including SCALE, scOpen, SAILER, peakVI, scCASE, and scDenoise. (a) Scatter plot of single cells in 2D space embedded on the raw and imputed Buenrostro2018 dataset. Each dot represents a single cell, and the color indicates its cell population. (b) Scatter plot of single cells in 2D space embedded on the raw and imputed GM12878/HL60 dataset. (c) Scatter plot of single cells in 2D space embedded on the raw and imputed InSilico dataset. (d) Scatter plot of single cells in 2D space embedded on the raw and imputed splenocyte dataset. (e) Heatmap of the confusion matrix by Hungarian algorithm on the raw and imputed Buenrostro2018 dataset.

scATAC-seq data exhibits high heterogeneity, the shifting of cellular heterogeneity is a hallmark of disorders. To examine the extent to which IMATAC and the other six competing methods, including SCALE, scOpen, SAILER, peakVI, scCASE, and scDenoise, alter the heterogeneity of individual cells, the individual cells were clustered by K-means on both the raw and imputed scATAC-seq data, and marked with the labels of cell populations to set off in contrast. For visualization of the results on the four experimental datasets, the peak count vector for each cell is embedded into a 2D space by combining Principal Component Analysis (PCA) and Uniform Manifold Approximation and Projection (UMAP) (Fig. 3a–d). Herein, each dot represents a single cell, and the color indicates its cell population. Generally, all the methods, especially IMATAC, SCALE, scOpen, peakVI, and scCASE, have a considerable ability to distinguish different cell types compared with that of raw data without imputation, although the clarity for these methods is different from each other. To further measure this ability, the clustering accuracy was scored by comparing the labels of clustering and the labels of cell populations using Adjusted Rand Index (ARI), Normalized Mutual Information (NMI), Adjusted Mutual Information (AMI). Generally, IMATAC can achieve higher accuracies except for that on Splenocyte dataset, where our proposed method seems weaker in some cases when standing against scOpen, peakVI, and scCASE (Supplementary Fig. S3). Furthermore, the clustering quality was also scored using Silhouette Coefficient (SC), which can be calculated without the labels of cell populations by considering both cohesion and separation of individual cells. It is gratifying that the coefficient of our IMATAC is always higher than that of the other methods on all the datasets apart from GM12878/HL60, which may further demonstrate the ability of our method to enhance cellular heterogeneity patterns. In addition, a confusion matrix was calculated on the imputed Buenrostro2018 dataset with the help of Hungarian algorithm, presenting an optimal one-to-one mapping of the labels between cell clustering and cell populations (Fig. 3e). It can be seen that IMATAC maximizes the sum of the diagonal in the matrix, demonstrating its power against that of the raw and the other six competing methods on this dataset. Meanwhile, the confusion matrices on the other three datasets can also be observed in Supplementary Fig. S4, and the comparative results are essentially consistent with those obtained using the first three clustering metrics. All the above suggest that our IMATAC is more conducive to retaining and enhancing the cluster patterns of single cells by recovering the dropout values.

### IMATAC benefits the differential analysis of chromatin accessibility

**Figure 4:**
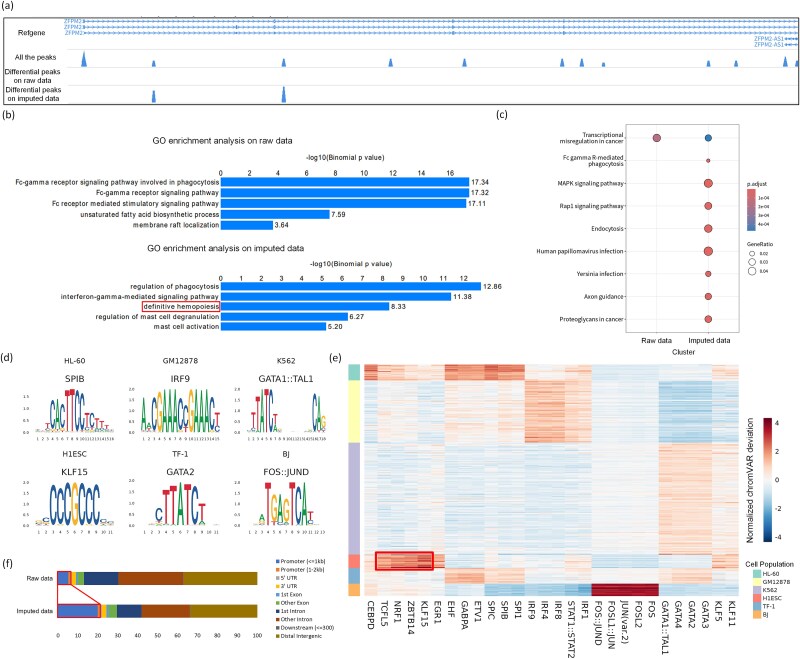
Biological significance of differential chromatin accessibility and cell-type-specific regulatory elements. (a) Comparison between the differential peaks detected on the raw and imputed scATAC-seq data. The differential peaks between two cell populations of HSCs and MPPs are detected on Buenrostro2018 dataset using Wilcoxon rank test. The peaks within a segment of chr8: 106304593–106880143 are visualized with the help of WashU epigenome browser. (b) the significant terms (p-value <0.05) given by GO enrichment analysis, which is conducted on the gene list that overlapped with the detected differential peaks. (c) Comparative results by pathway analysis, which is also conducted on the gene list that overlapped with the detected differential peaks. (d) Transcription factor motifs that are most highly enriched in six different cell populations. These motifs are identified by Signac toolkit on the imputed InSilico dataset with the help of JASPAR database. (e) Heatmap of the accessibility variations of the top five motifs for each of the six cell populations. The accessibility variations were scored by chromVAR. (f) Comparison of peak annotations for MEPs between the raw and imputed Buenrostro2018 dataset.

To investigate how the proposed IMATAC affects the downstream differential analysis of chromatin accessibility, the differential peaks between two cell populations of HSCs and MPPs were identified on the imputed Buenrostro2018 dataset using Wilcoxon rank test from scATAC-pro [42], and compared with those detected on the raw data without imputation. First of all, the genes that overlapped with the differential peaks on genome were listed using ChIPseeker [43], and visualized with the help of WashU Epigenome Browser [44]. The results tell that our imputation procedure is more conducive to the detection of biologically meaningful peaks. For example, a series of differential peaks were detected in the region of gene *ZFPM2* on the imputed data, this gene is known as an extensively characterized cofactor of hematopoietic transcription [45]. In contrast, no differential peaks can be found in the same region on the raw data (Fig. 4a). Besides, a similar issue was also observed in the region of gene *CDC5L*, which is abundantly expressed in bone marrow cells serving as a critical cell cycle regulator essential for the G2/M transition [46], and there are more differential peaks detected on imputed data compared with those on raw data (Supplementary Fig. S5a). Furthermore, a Gene Ontology (GO) enrichment analysis was tried using GREAT [47] on the gene list that overlapped with the differential peaks (Fig. 4b). We found that some enriched GO terms (*p*-value <0.05) with obvious biological meaning can be discerned on the imputed data, such as Definitive Hemopoiesis (GO:0060216), which occurs primarily in bone marrow and involves hierarchical differentiation processes starting from HSCs to MPPs and the other blood cell types [48]. But this term fails to be discerned on the raw data without imputation. In addition, a set of Kyoto Encyclopedia of Genes and Genomes (KEGG) pathway analysis was also conducted in the same way using ReactomePA [49] and clusterProfiler [50]. It can be seen that there are differences between the results on the raw and imputed data (Fig. 4c). Especially, a Mitogen-Activated Protein Kinase (MAPK) signaling pathway can be detected on the imputed data rather than raw data. This signaling pathway of MAPK is known to play a critical role in proliferation, differentiation, and apoptosis of hematopoietic progenitor cells from CD34+ human bone marrow [51, 52]. It tells that our imputation approach helps to gain insights into the biological implications of the changes in chromatin accessibility.

### IMATAC benefits the discovery of regulatory elements

To examine whether our proposed IMATAC benefits the discovery of regulatory elements by means of imputation, a differential accessibility region analysis was carried out using Signac [53] toolkit on both raw and imputed scATAC-seq data. There are 6513 significant cell-type-specific accessible peaks (*p*-adjust value <0.05) that can be identified from a total of 89 610 peaks for cell population MEPs on the imputed Buenrostro2018 dataset, which is much more than the 3210 ones on the raw data. The same trend can also be observed on the other datasets, although not always that obvious, such as 24 807 versus 24 914 for all the six cell populations on the raw and imputed InSilico dataset. Taking a step forward, the transcription factor (TF) binding motifs within these cell-type-specific peaks were investigated with the help of JASPAR database [54]. The top five TF motifs with the smallest *p*-value for each of the six imputed cell populations were extracted (Supplementary Fig. S5b), and the most highly enriched one was shown (Fig. 4d). Among them, the transcription factor IRF9 gets the highest enrichment in GM12878, and the IRF family TFs were reported to be crucial for the pathogenesis of lymphoma [55]. The transcription factor GATA1::TAL1 is most highly enriched in K562, which is derived from the human myeloid leukemia cell line and can differentiate into erythroid cells, and the binding of GATA1 and TAL1 was found to regulate normal myeloid differentiation and may contribute to malignant transformation in blood cells [56]. This kind of correlation can also be seen for the imputed cell population MEPs (Supplementary Fig. S5c), where the enriched transcription factor KLF4 acts as a reprogramming factor in the generation of myeloid blood cells [57]. Besides, the accessibility variations of the top five motifs for each of the six imputed cell populations were scored by chromVAR (Fig. 4e). The transcription factor NRF1 presents a considerably high activity for cell population H1ESC, which is believed to play an important role in the inhibition of stem cell apoptosis [58]. In addition, we tried to annotate the cell-type-specific accessible peaks with the possible *cis-*regulatory elements using ChIPseeker [43]. It is shown that only 6.2% of the 3210 cell-type-specific accessible peaks from the raw data for MEPs were annotated as promoters, in contrast, this proportion is increased to 21.3% while examining the 6513 ones from the imputed data (Fig. 4f). More importantly, some peaks that are annotated as promoters on the imputed data but not on the raw data may have biological meanings. For example, on the imputed data, the peak located at chr21: 44524601–44524988 was annotated as the promoter region for gene *U2AF1*, which is highly expressed in hematopoietic progenitor cells [59], but this region will be missed without imputation. That may demonstrate the potential of our IMATAC to recover the *cis-*regulatory elements to some extent.

## Discussion

### Hierarchical network benefits recovery of dropout events

**Figure 5:**
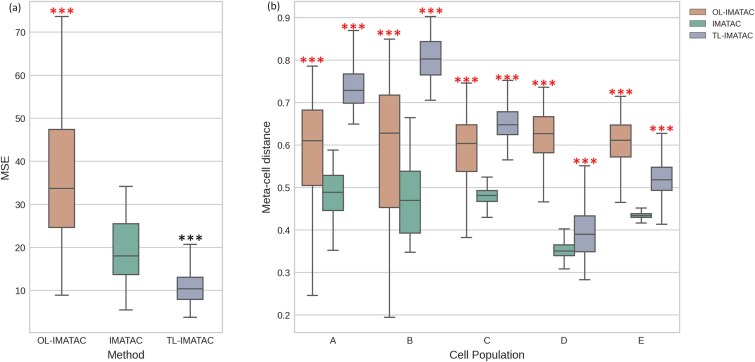
Comparison of the performance between IMATAC and its variants with different levels of hierarchical autoencoders. There are two variants being involved, one with one level and the other with three levels, which are noted as OL-IMATAC and TL-IMATAC, respectively. The comparison is conducted on the simulated scATAC-seq data of five cell types (a, B, C, D, and E) at a masking rate of 40%. The statistical significance is calculated using a one-tailed t-test, the ranges of p-values are marked with different numbers of asterisks considering the limited space, i.e. one asterisk for [0.05, 0.01), two asterisks for [0.01, 0.001), and three for [0.001, −∞), and the directions are separately indicated by red and black colors. (a) Box plot of imputation error in terms of MSE by OL-IMATAC, IMATAC, and TL-IMATAC. (b) Box plot of imputation error in terms of meta-cell distance by OL-IMATAC, IMATAC, and TL-IMATAC.

Considering the high dimensionality and sparsity of scATAC-seq data, IMATAC is designed to have a hierarchical autoencoder with two levels, including bottom level and top level. The former is dedicated to capturing the local details, while the latter focuses on profiling the global features. To discuss the advantages of this hierarchical network architecture involved in IMATAC, we tried to compare our method with its two variants with different levels of hierarchical autoencoders, one with one level and the other with three levels, which are noted as OL-IMATAC and TL-IMATAC, respectively. In the implementation, the corrupted version of the simulated scATAC-seq data of five cell types (A, B, C, D, and E) at a masking rate of 40% was separately fed into OL-IMATAC, IMATAC, and TL-IMATAC, and their performances are compared with each other in terms of MSE (Fig. 5a) and meta-cell distance (Fig. 5b). For MSE, our IMATAC has medium values, which are superior to those of OL-IMATAC but not as good as those of TL-IMATAC. In other words, the more hierarchical levels the autoencoder has, the smaller the value of MSE will be. This substantially makes sense since an autoencoder with a higher level can better extract features from data on multiple scales, which is beneficial for the embedding and reconstruction of high-dimensional sparse scATAC-seq data. When it comes to meta-cell distance, the results are not exactly the same. Our two-level IMATAC can achieve the smaller distances even standing against TL-IMATAC, which is most likely attributed to the fact that the autoencoder with three levels cannot always enhance the classification of individual cells while improving the reconstruction of data. Thus, it is believed that the hierarchical architecture in IMATAC benefits the recovery of missing data by dropout events, due to its comprehensive characterization of high-dimensional sparse scATAC-seq data.

### Unveiling the contributions of network modules

**Figure 6:**
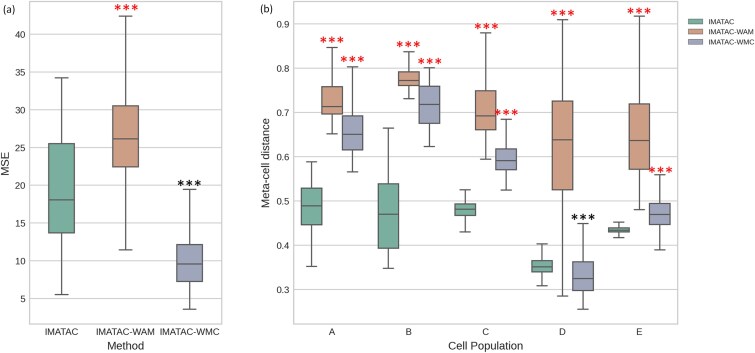
Comparison of the performance between IMATAC and its variants without each of the two network modules, including artificial masking and multi-classifier. There are two variants being involved, one without artificial masking and the other without multi-classifier, which are noted as IMATAC-WAM and IMATAC-WMC, respectively. The ablation experiments were also conducted on the simulated scATAC-seq data of five cell types (A, B, C, D, and E) at a masking rate of 40%. The statistical significance is calculated using a one-tailed t-test, the ranges of p-values are marked with different numbers of asterisks considering the limited space, i.e. one asterisk for [0.05, 0.01), two asterisks for [0.01, 0.001), and three for [0.001, −∞), and the directions are separately indicated by red and black colors. (a) Box plot of imputation error in terms of MSE by IMATAC, IMATAC-WAM, and IMATAC-WMC. (b) Box plot of imputation error in terms of meta-cell distance by IMATAC, IMATAC-WAM, and IMATAC-WMC.

Apart from the hierarchical network, the denoising autoencoder and dual-task learning are also regarded as the contributions of our IMATAC. Thus, it seems meaningful to discuss how these network modules affect the effectiveness of our proposed method. To this end, two ablation experiments were separately conducted in terms of artificial masking and multi-classifier on the simulated data following the same way as that of the hierarchical network above (Fig. 6), where the network variants without artificial masking and multi-classifier are noted as IMATAC-WAM and IMATAC-WMC, respectively. It can be observed that the removal of artificial masking from IMATAC causes significantly greater MSE (*p*-value ≤0.001) and meta-cell distance (*p*-value ≤0.001). That is to say, the denoising autoencoder is beneficial for the network to recover the missing values and enhance the heterogeneous patterns of single cells, since it encourages our method to learn to reconstruct the original data from an artificially corrupted version, and the recovery of missing values helps single cells to achieve a high correlation with their meta-cell. In contrast, the absence of multi-classifier yields smaller MSE errors (*p*-value ≤0.001). It may be because the dual-task learning that considers both reconstruction and classification has been reduced to a single-task learning that only focuses on reconstruction, which is also reflected in the loss function where MSE stays while cross-entropy is excluded accordingly. As for meta-cell distance, the removal of multi-classifier leads to an increase for all five cell types except for D, which is in line with our expectations considering that the learning of our network is no longer constrained by cell populations in this configuration. Generally, these two modules contribute to the imputation of our IMATAC.

### Separation of the dropout zeros from biological zeros

At first glance, an imputation method is to assign non-zero values to some zero elements of a scATAC-seq matrix. But in fact, simply altering the counts of a large number of peaks with zero values is not necessarily a good strategy for the imputation method. Previous studies have shown that preserving more biological zeros in single-cell data actually benefits the downstream analyses [60]. Thus, we discuss how well our IMATAC can separate the zeros by dropout events from the zeros with biological meaning. To do it, the simulated scATAC-seq data of five cell types (A, B, C, D, and E) is regarded as ground truth, and its corrupted version with a portion of peaks masked for each cell was fed into IMATAC for imputation. At a masking rate of 40%, there are a total of 5 635 799 zeros by dropout events, and 75.70% of them can be successfully recovered. At the same time, up to 97.59% of the 177 421 801 zeros with biological meaning can be retained. Besides, at a masking rate of 60%, there are a total of 7 513 637 dropout zeros and 175 543 963 biological zeros, and 75.67% and 99.01% of them can be recovered and retained, respectively. It suggests that our IMATAC has a considerable ability to impute the missing values by dropout events in scATAC-seq data, while preserving most biological zeros unchanged. This advantage may be attributed to its dual-task learning strategy, where IMATAC tries to learn to reconstruct the original scATAC-seq data primarily relying on the cells under the same population.

**Key Points**

- We propose IMATAC, a deep hierarchical network with denoising autoencoder for the imputation of scATAC-seq data in the form of peak by cell.
- IMATAC learns to reconstruct the original scATAC-seq data from an artificially corrupted version through a hierarchical denoising autoencoder, so as to acquire an ability to recover dropout values primarily relying on the cells under the same populations with the help of a parallel multi-classifier.
- IMATAC can achieve lower imputation errors, and benefit the downstream analysis, including heterogeneous clustering and differential analysis.
- We provide an efficient and well-documented Python package for IMATAC, which is available at https://github.com/lhqxinghun/IMATAC.

## Supplementary Material

Supplementary_File_bbaf515

## Data Availability

The sources for all the scATAC-seq data involved in this study can be found in Supplementary File. The source code is available at https://github.com/lhqxinghun/IMATAC, including a Python package IMATAC as well as the scripts for simulation, imputation, and evaluation.
