# Supplementary material for: IMATAC imputes single-cell ATAC-seq data by deep hierarchical network with denoising autoencoder
Source: Brief Bioinform. 2025 Sep 29;26(5):bbaf515. doi: 10.1093/bib/bbaf515 (PMC12478030; doi:10.1093/bib/bbaf515)
Supplement: Supplementary_File_bbaf515 [file supplementary_file_bbaf515.docx]

Supplementary File for

**IMATAC imputes single-cell ATAC-seq data by deep hierarchical network with denoising autoencoder**

Yao Li^1^, Hongqiang Lyu^1^*, Kexin Li^1^, Xinman Zhang^1^, Yuan Liu^1^, Pengcheng Jing^1^, Ze Liu^2^, Peng Han^3^*

^1^School of Automation Science and Engineering, Faculty of Electronic and Information Engineering, Xi’an Jiaotong University, Shaanxi 710049, China

^2^College of Water Resources and Architectural Engineering, Northwest A&F University, Shaanxi 712100, China

^3^Department of Otorhinolaryngology-Head and Neck Surgery, The First Affiliated Hospital, Xi’an Jiaotong University, Shaanxi 710061, China

^*^Correspondence and requests for materials should be addressed to H.L. (email: [hongqianglv@mail.xjtu.edu.cn](mailto:hongqianglv@mail.xjtu.edu.cn))

**Contents：**

I. Supplementary datasets

II. Supplementary methods

III. Supplementary Tables

IV. Supplementary Figures

V. Supplementary Reference

**I. Supplementary datasets**

**Supplementary Table S1** Simulated and experimental scATAC data involved in the paper.

| Type | Dataset | Number of cells | Number of peaks | Number of cell populations | Population name | URL |
| --- | --- | --- | --- | --- | --- | --- |
| Simulated | Cell type A, B, C, D, and E | 2,500 | 80,000 | 5 | A, B, C, D, and E | Generated by simCAS [1] at https://github.com/Chen-Li-17/simCAS |
| Experimental | Buenrostro2018 | 2,034 | 89,610 | 10 | HSCs, MPPs, LMPPs, CMPs, GMPs, MEPs, CLPs, mono, UNKs, pDCs | https://www.dropbox.com/sh/8o8f0xu6cvr46sm/AAB6FMIDvHqnG6h7athgcm5-a/Buenrostro_2018.tar.gz?dl=0 |
| Experimental | GM12878/HL60 | 1,987 | 125,289 | 2 | GM12878, HL60 | https://www.ncbi.nlm.nih.gov/geo/query/acc.cgi?acc=GSM2970932 |
| Experimental | InSilico | 1,377 | 68,069 | 6 | K562, GM12878, HL-60, BJ, TF-1, H1ESC | https://www.ncbi.nlm.nih.gov/geo/query/acc.cgi?acc=GSE65360  or  https://zenodo.org/records/3984189#.XzDpJRNKhTY |
| Experimental | Splenocyte | 3,166 | 77,294 | 12 | Naive_CD8_T, Transitional_B, Marginal_Zone_B, Naive_CD4_T, Follicular_B, Granulocyte, Dendritic_cell, Regulatory_T, CD27+_Natural_Killer, Memory_CD8_T, Macrophage, CD27-_Natural_Killer | https://www.ebi.ac.uk/biostudies/ArrayExpress/studies/E-MTAB-6714?query=E-MTAB-6714  or  https://zenodo.org/records/3984189#.XzDpJRNKhTY |

**II. Supplementary methods**

**Data simulation**

For data simulation, a state-of-the-art embedding-based method for simulating single-cell chromatin accessibility sequencing data, called simCAS, is employed to generate peak by cell matrix under different cell populations. In the implementation, simCAS was running in pseudo-cell-type mode, the Buenrostro2018 dataset was fed into it for parameter estimation, and peak by cell matrix with population label for each cell can be produced. Besides, a total of six scATAC-seq datasets with other considerations were also produced, including different levels of noise (level 1, level 2, and level 3) and different degrees of sparsity (0.4, 0.5, and 0.6). In the implementation, the noise was randomly sampled from a normal distribution $N\sim(0, 1)$, and scaled at three different ratios. Then the noise was added to each element of the simulated matrix, and the sum was rounded off. For the sparsity, a parameter named zero_prob in simCAS is set to 0.4, 0.5 (by default), and 0.6, respectively, and the simulated data with different sparsity can be produced. Using the simulated data above, we performed an artificial mask to mimic the dropout events that may occur during biological experiments. The artificial mask was created using a Bernoulli binomial distribution, which determines the likelihood of each peak being masked based on a specified masking rate, including 20%, 40%, and 60%. This approach allows us to simulate various dropout events that might be encountered in real biological datasets. The simulated data without artificial masking serves as the ground truth, while the data with artificial masking is fed into different methods for imputation. It is worth noting that the simulated data with different levels of noise (level 1, level 2, and level 3) and different degrees of sparsity (0.4, 0.5, and 0.6) were only masked at a rate of 40%.

**Clustering analysis**

For scATAC-seq data in the form of peak by cell matrix, Principal Component Analysis (PCA) is first used to reduce the peak count vector for each cell to 1,000 dimensions. After that, the first principal component PC1 is removed, and Uniform Manifold Approximation and Projection (UMAP) is employed to further reduce its dimensionality to 200. Then K-means algorithm is used to cluster single cells with extracted features by PCA and UMAP. Further UMAP is conducted to reduce the dimension to 2, so that the scatter plot of individual cells in two-dimensional space is obtained.

**Evaluation Metrics**

*Meta-cell distance*: Given the ground truth matrix $\boldsymbol{X}\in\mathbb{R}^{m\times n}$ and the imputed matrix $\text{Y}\in\mathbb{R}^{m\times n}$, the meta-cell distance between the imputed data and the ground truth data can be calculated. Firstly, a meta-cell is computed as the average of cells belonging to the same cell population $k$:

$X_{meta}^{k}=\frac{\sum_{j=1}^{n^{k}} X_{\cdot j}^{k}}{n^{k}}$, (1)

where $n^{k}$ is the number of single cells under population $k$, $X_{\cdot j}^{k}$ denotes the peak count vector for the $j_{th}$ cell, that is, the $j_{th}$ column in peak by cell matrix $\boldsymbol{X}$**,** and the cell is under population $k$. Then a meta-cell distance between the imputed peak count vector for the $j_{th}$ cell and its corresponding meta-cell $X_{meta}^{k}$ is given out using Bray-Curtis dissimilarity:

$D_{j}^{k}=\mathrm{BCd}(Y_{\cdot j}^{k},X_{meta}^{k})$, (2)

where $D_{j}^{k}$ is the meta-cell distance for the $j_{th}$ cell, the cell is under population $k$, and $\mathrm{BCd}$ is the function of Bray-Curtis dissimilarity. A smaller distance indicates a better imputation.

*Adjusted Rand Index*: Adjusted Rand Index (ARI) is a statistical measure used to assess the similarity between two clustering, adjusting for chance. It is improved upon the Rand Index (RI) by accounting for random agreements, providing a more reliable validation metric. Given true labels $T$ and clustering labels L, Rand Index is defined as:

$\text{RI}\text{ = }\frac{a+b}{C_{N}^{2}}$, (3)

where $a$ is the number of element pairs that are in the same cluster across $T$ and $L$, $b$ is the number of element pairs that are in the different clusters across $T$ and $L$, $C_{N}^{2}$ denotes the total number of pairs across $T$ and $L$. Considering that Rand Index has a bias towards high values, even for random clustering, so that the Adjusted Rand Index (ARI) is formulated:

$\text{ARI}\text{ = }\frac{RI-\text{Expected}\text{ }\text{(}RI)}{\max(RI)-\text{Expected }\text{(}RI)}$, (4)

*Normalized mutual information*: Normalized Mutual Information (NMI) is another metric for evaluation of the similarity between two clustering. NMI is defined as:

$\text{NMI}=\frac{2\times I(T,L)}{H(T)+H(L)}$, (5)

where $I(T,L)$ is the mutual information between true labels $T$ and clustering labels $L$, while $H(T)$ and $H(L)$ are the entropy of $T$ and *L*, respectively.

*Adjusted Mutual Information*: Adjusted Mutual Information (AMI) is another metric for the evaluation of the similarity between two clustering while correcting for chance. It is built on Mutual Information (MI) but adjusts for the possibility of random agreement between the two clustering. That makes AMI particularly useful while comparing clustering with different sizes or while the number of clusters varies. AMI is defined as:

$\text{AMI}=\frac{I(T,L)-E[I(T,L)]}{max[H(T),H(L)]-E[I(T,L)]}$, (6)

where $E[I(T,L)]$ is the expected mutual information between true labels $T$ and clustering labels $L$ under an assumption of random cluster assignments.

**Competing methods**

*SCALE:* SCALE [2] is a computational method for analyzing scATAC-seq data, which combines a deep generative framework and a probabilistic Gaussian Mixture Model to learn latent features that accurately characterize scATAC-seq data. To implement this method, the source code was downloaded from <https://github.com/jsxlei/SCALE>.

*scOpen:* scOpen [3] is a computational method designed to address the sparsity issue in scATAC-seq data by employing regularized non-negative matrix factorization and TF-IDF transformation for imputing and quantifying open chromatin states in regulatory regions. The source code of scOpen was downloaded from <https://github.com/CostaLab/scopen>.

*SAILER:* SAILER [4] is a deep generative model framework for analyzing scATAC-seq data, which adopts a variational autoencoder to learn the latent representation, but imposes additional constraints to ensure the independence of the learned representations from the confounding factors, including read depth and batch effects. The source code of SAILER was downloaded from https://github.com/uci-cbcl/SAILER.

*peakVI:* peakVI [5] is a probabilistic framework that leverages deep neural networks to analyze scATAC-seq data, which fits an informative latent space that preserves biological heterogeneity while correcting batch effects and accounting for technical effects, such as library size and region-specific biases. It pays more attention to differential analysis rather than imputation. The source code of peakVI was downloaded from https://github.com/scverse/scvi-tools.

*scCASE:* scCASE [6] is a scATAC-seq data enhancement method, which employs non-negative matrix factorization with an iteratively updated cell-to-cell similarity matrix to improve data quality and interpretability. The source code of scCASE was downloaded from <https://github.com/BioX-NKU/scCASE>.

*scDenoise:* scDenoise [7] is a reference-based scRNA-seq denoising method using semi-supervised learning, which is dedicated to denoising unlabeled target data based on annotated cells in the high-quality reference datasets, utilizing biological characteristics hidden in these datasets. The source code of scDenoise was downloaded from https://github.com/zhongfqi/SCdenoise.

**III. Supplementary Tables**

**Supplementary Table S2** Imputation error in term of MSE by IMATAC and the other three methods.

| Masking rate 20% | | | | | | | |
| --- | --- | --- | --- | --- | --- | --- | --- |
|  | IMATAC | SCALE | scOpen | SAILER | peakVI | scCASE | scDenoise |
| MSE | 14.89±5.88 | 35.18±14.23 | 41.53±9.74 | 47.10±3.63 | 77.74±7.54 | 130.84±31.51 | 37.44±8.26 |
| Masking rate 40% | | | | | | | |
|  | IMATAC | SCALE | scOpen | SAILER | peakVI | scCASE | scDenoise |
| MSE | 19.63±7.78 | 36.24±14.67 | 39.51±8.95 | 50.03±4.10 | 75.67±6.70 | 128.86±28.29 | 37.14±7.78 |
| Masking rate 60% | | | | | | | |
|  | IMATAC | SCALE | scOpen | SAILER | peakVI | scCASE | scDenoise |
| MSE | 16.57±6.14 | 32.12±12.32 | 37.03±7.98 | 48.86±3.65 | 70.46±5.85 | 126.00±26.91 | 36.76±7.46 |

**Supplementary Table S3** Imputation error in term of meta-cell distance by IMATAC and the other three methods.

| Masking rate 20% | | | | | | | |
| --- | --- | --- | --- | --- | --- | --- | --- |
| Cell population | IMATAC | SCALE | scOpen | SAILER | peakVI | scCASE | scDenoise |
| A | 0.42±0.04 | 0.48±0.13 | 0.92±0.02 | 0.92±0.01 | 0.84±0.01 | 0.93±0.02 | 0.67±0.07 |
| B | 0.44±0.05 | 0.47±0.16 | 0.91±0.02 | 0.94±0.00 | 0.88±0.01 | 0.94±0.02 | 0.76±0.06 |
| C | 0.44±0.01 | 0.50±0.09 | 0.93±0.02 | 0.90±0.01 | 0.80±0.01 | 0.88±0.02 | 0.54±0.04 |
| D | 0.35±0.02 | 0.50±0.07 | 0.95±0.02 | 0.79±0.01 | 0.72±0.03 | 0.87±0.02 | 0.30±0.05 |
| E | 0.40±0.01 | 0.51±0.06 | 0.94±0.02 | 0.86±0.01 | 0.74±0.01 | 0.89±0.02 | 0.45±0.04 |
| Masking rate 40% | | | | | | | |
| Cell population | IMATAC | SCALE | scOpen | SAILER | peakVI | scCASE | scDenoise |
| A | 0.48±0.06 | 0.49±0.14 | 0.92±0.01 | 0.91±0.01 | 0.83±0.01 | 0.94±0.01 | 0.67±0.05 |
| B | 0.47±0.08 | 0.48±0.16 | 0.91±0.02 | 0.94±0.01 | 0.88±0.01 | 0.94±0.02 | 0.75±0.05 |
| C | 0.48±0.02 | 0.50±0.10 | 0.93±0.02 | 0.89±0.01 | 0.79±0.01 | 0.88±0.01 | 0.54±0.02 |
| D | 0.35±0.02 | 0.51±0.07 | 0.96±0.02 | 0.78±0.01 | 0.70±0.04 | 0.86±0.02 | 0.33±0.04 |
| E | 0.44±0.01 | 0.52±0.06 | 0.94±0.02 | 0.85±0.01 | 0.73±0.02 | 0.89±0.02 | 0.46±0.04 |
| Masking rate 60% | | | | | | | |
| Cell population | IMATAC | SCALE | scOpen | SAILER | peakVI | scCASE | scDenoise |
| A | 0.44±0.05 | 0.44±0.14 | 0.93±0.01 | 0.91±0.01 | 0.82±0.01 | 0.93±0.02 | 0.67±0.05 |
| B | 0.45±0.07 | 0.45±0.16 | 0.92±0.02 | 0.94±0.00 | 0.87±0.01 | 0.94±0.02 | 0.76±0.05 |
| C | 0.45±0.01 | 0.45±0.10 | 0.94±0.01 | 0.89±0.01 | 0.77±0.01 | 0.87±0.03 | 0.54±0.02 |
| D | 0.35±0.02 | 0.44±0.08 | 0.96±0.00 | 0.78±0.01 | 0.68±0.04 | 0.85±0.03 | 0.33±0.03 |
| E | 0.40±0.01 | 0.46±0.06 | 0.95±0.01 | 0.85±0.01 | 0.71±0.02 | 0.89±0.02 | 0.47±0.03 |

**IV. Supplementary Figures**


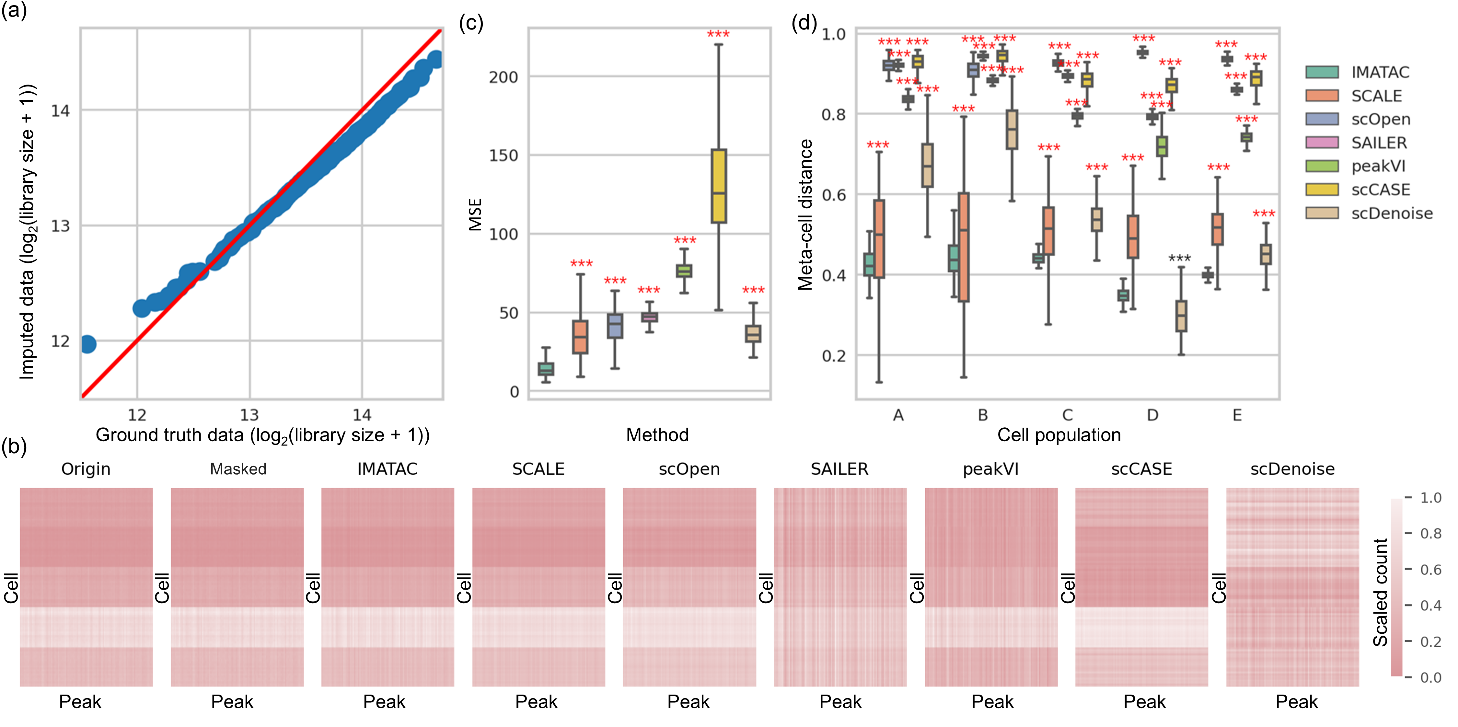


**Supplementary Figure S1 Results on simulated scATAC-seq data.** The performance of IMATAC is investigated on simulated scATAC-seq data of five cell types (A, B, C, D, and E) at a masking rate of 20%, and compared with that of the other six competing methods, including SCALE, scOpen, SAILER, peakVI, scCASE, and scDenoise. (a) Scatter plot of library size between ground truth data and imputed data by IMATAC. (b) Heatmaps of peak by cell matrices, including original, masked, and imputed by IMATAC and the other six methods. The high-dimensional matrices have been reduced for visualization by merging surrounding counts. (c) Box plot of imputation error in terms of MSE by IMATAC and the other six methods. (d) Box plot of imputation error in terms of meta-cell distance by IMATAC and the other six methods.


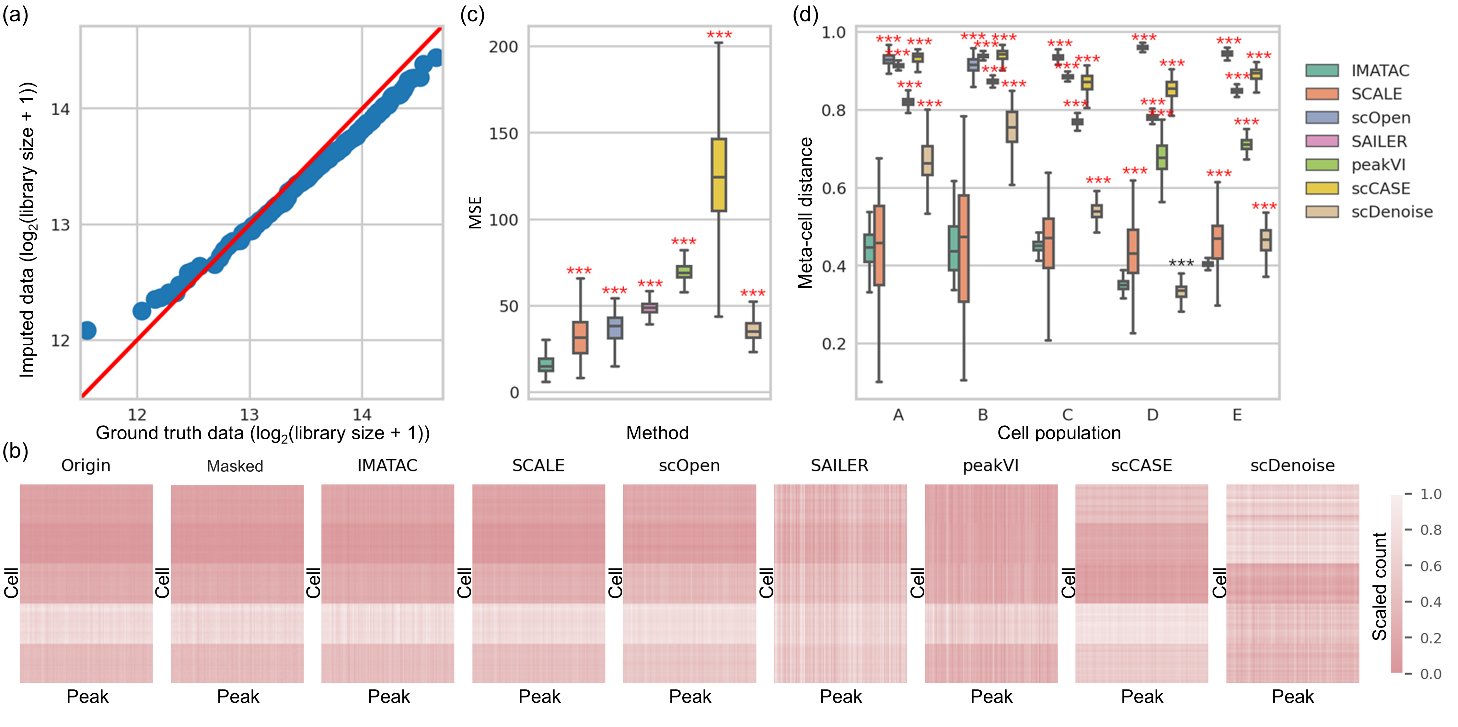


**Supplementary Figure S2 Results on simulated scATAC-seq data.** The performance of IMATAC is investigated on simulated scATAC-seq data of five cell types (A, B, C, D, and E) at a masking rate of 60%, and compared with that of the other six competing methods, including SCALE, scOpen, SAILER, peakVI, scCASE, and scDenoise. (a) Scatter plot of library size between ground truth data and imputed data by IMATAC. (b) Heatmaps of peak by cell matrices, including original, masked, and imputed by IMATAC and the other six methods. The high-dimensional matrices have been reduced for visualization by merging surrounding counts. (c) Box plot of imputation error in terms of MSE by IMATAC and the other six methods. (d) Box plot of imputation error in terms of meta-cell distance by IMATAC and the other six methods.


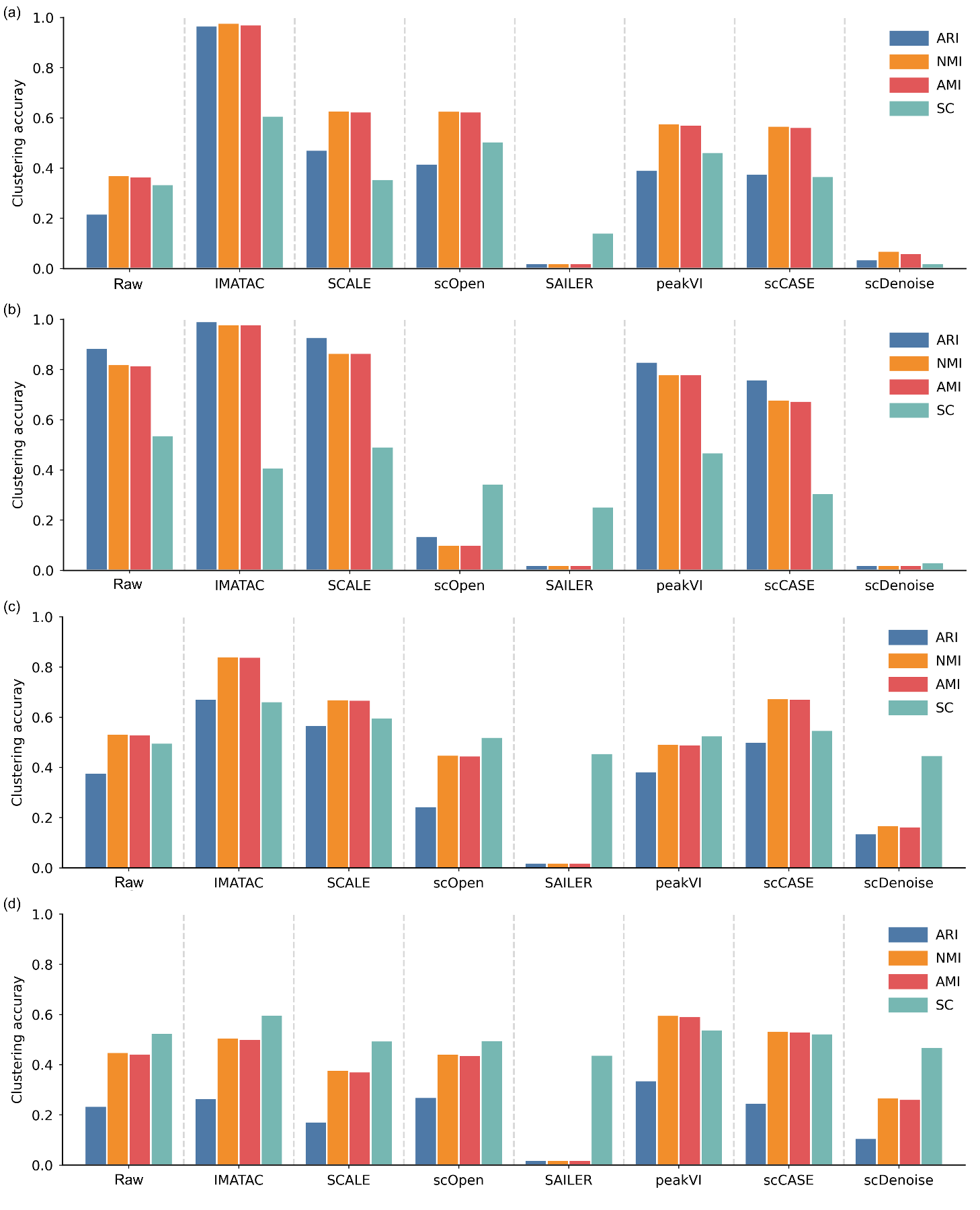


**Supplementary Figure S3 Clustering accuracy of imputed scATAC-seq data.** Single cells are embedded by combining PCA and UMAP, and clustered using K-means on scATAC-seq data imputed by IMATAC and the other six competing methods, including SCALE, scOpen, SAILER, peakVI, scCASE, and scDenoise. (a) Histogram of ARI, NMI, AMI, and SC on raw and imputed Buenrostro2018 dataset. (b) Histogram of ARI, NMI, AMI, and SC on raw and imputed GM12878/HL60 dataset. (c) Histogram of ARI, NMI, AMI, and SC on raw and imputed InSilico dataset. (d) Histogram of ARI, NMI, AMI, and SC on raw and imputed Splenocyte dataset.


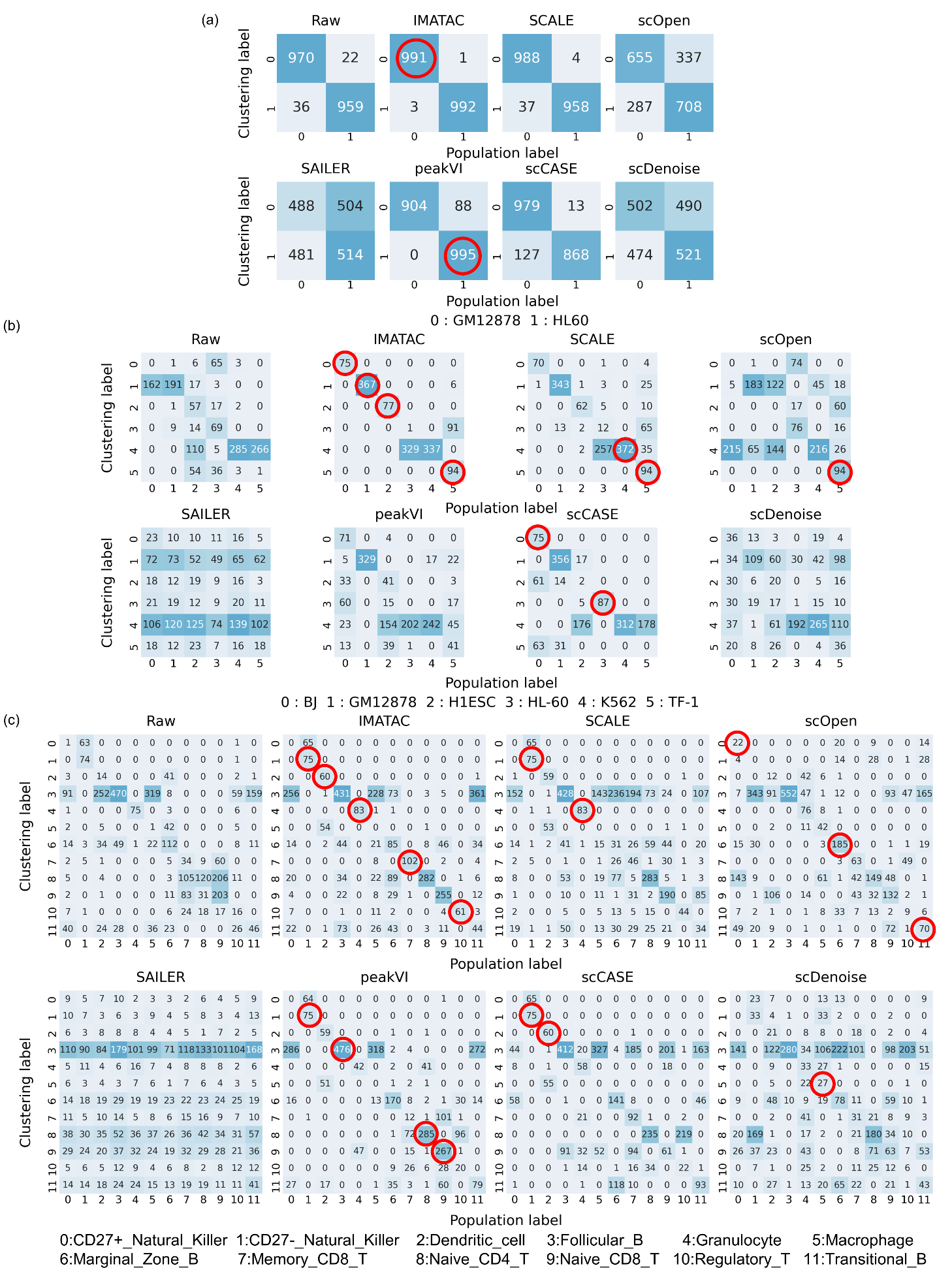
**Supplementary Figure S4 Confusion matrix of imputed scATAC-seq data.** Single cells are embedded by combining PCA and UMAP, and clustered using K-means on scATAC-seq data imputed by IMATAC and the other six competing methods, including SCALE, scOpen, SAILER, peakVI, scCASE, and scDenoise. (a) Heatmap of the confusion matrix by Hungarian algorithm on raw and imputed GM12878/HL60 dataset. (b) Heatmap of the confusion matrix by Hungarian algorithm on raw and imputed InSilico dataset. (c) Heatmap of the confusion matrix by Hungarian algorithm on raw and imputed Splenocyte dataset.


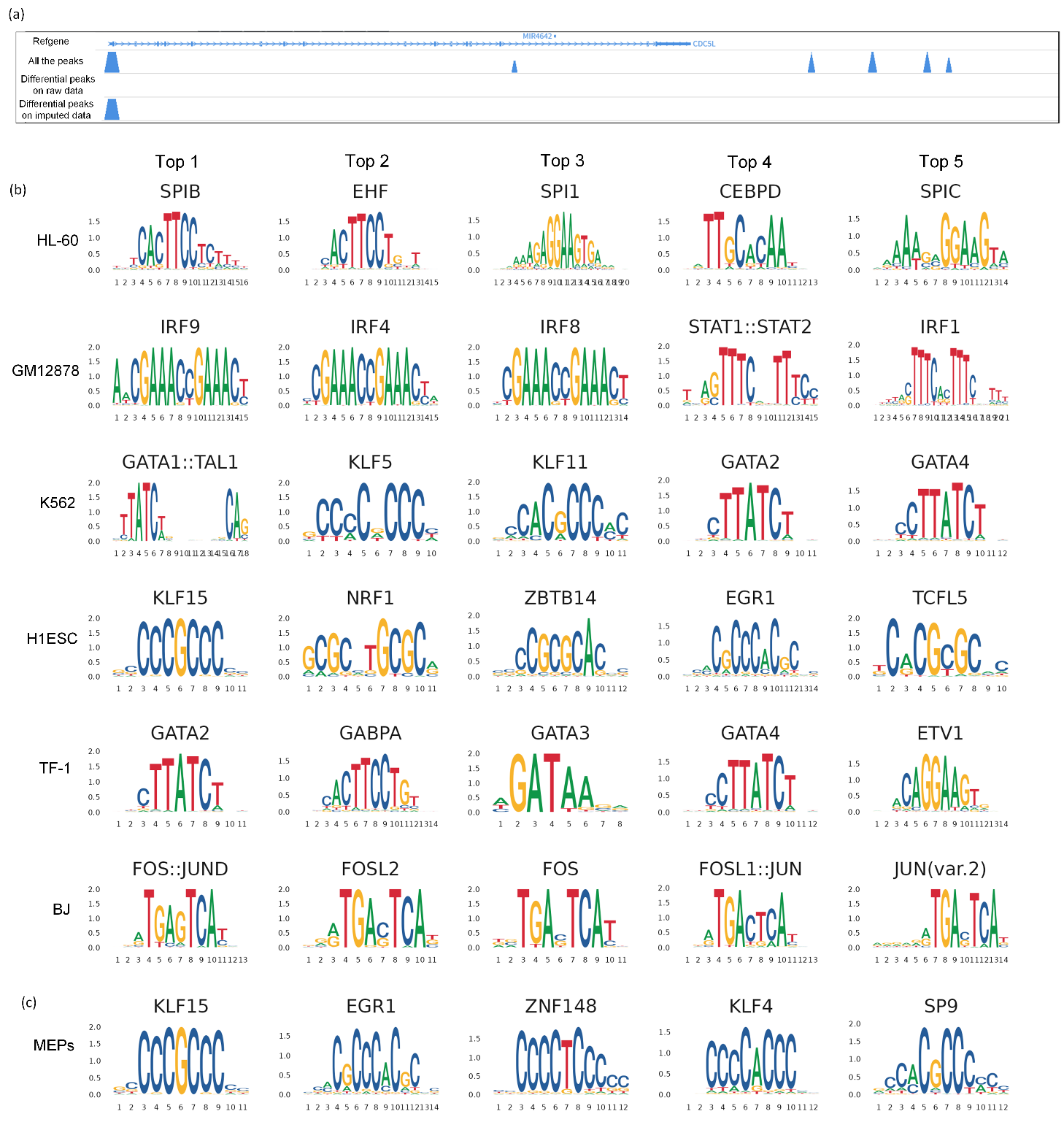


**Supplementary Figure S5 Biological significance of differential chromatin accessibility and transcription factor motif enrichment.** (a) Comparison between the differential peaks detected on the raw and imputed scATAC-seq data. The differential peaks between two cell populations of HSCs and MPPs are detected on Buenrostro2018 dataset using Wilcoxon rank test. The peaks within a segment of chr6: 44345832-44921382 are visualized with the help of WashU Epigenome Browser. (b) Top five transcription factor motifs that are enriched in different cell populations. These motifs are identified by Signac toolkit on the imputed Insilico dataset with the help of JASPAR database. (c) Top five transcription factor motifs that are enriched in different cell populations. These motifs are identified for MEPs by Signac toolkit on the imputed Buenrostro2018 dataset with the help of JASPAR database.

**V. Supplementary Reference**

1. Li C, Chen X, Chen S et al. simCAS: an embedding-based method for simulating single-cell chromatin accessibility sequencing data, Bioinformatics 2023;39:btad453.

2. Xiong L, Xu K, Tian K et al. SCALE method for single-cell ATAC-seq analysis via latent feature extraction, Nature communications 2019;10:4576.

3. Li Z, Kuppe C, Ziegler S et al. Chromatin-accessibility estimation from single-cell ATAC-seq data with scOpen, Nature communications 2021;12:6386.

4. Cao Y, Fu L, Wu J et al. SAILER: scalable and accurate invariant representation learning for single-cell ATAC-seq processing and integration, Bioinformatics 2021;37:i317-i326.

5. Ashuach T, Reidenbach DA, Gayoso A et al. PeakVI: A deep generative model for single-cell chromatin accessibility analysis, Cell Reports Methods 2022;2:100182.

6. Tang S, Cui X, Wang R et al. scCASE: accurate and interpretable enhancement for single-cell chromatin accessibility sequencing data, Nature communications 2024;15:1629.

7. Zhong F, Zeng Y, Liu Y et al. SCdenoise: a reference-based scRNA-seq denoising method using semi-supervised learning. In: 2022 IEEE International Conference on Bioinformatics and Biomedicine (BIBM). 2022, p. 182-185.
